# A SYSTEMATIC REVIEW OF THE ART-BASED RESEARCH WITH OLD ADULTS IN SOUTH KOREA

**DOI:** 10.1093/geroni/igad104.3154

**Published:** 2023-12-21

**Authors:** Yeojin Yoon

**Affiliations:** Chung-Ang University, Seoul, Republic of Korea

## Abstract

This study aims to review and summarize the literature on the use of Arts-based Research(ABR) with older adults in South Korea. The study was conducted as a systematic literature review. ABR is method that utilizes the arts to expand horizons of awareness. In South Korea, there is a large gap between the literacy levels of young and older adults and the differences in their cultures. Therefore, using art to convey expressions that are difficult to communicate through text and utilizing common values through art can be more effective than using text. This study was designed using Electronic databases. Keywords for older adult and ABR were searched in Electronic databases, and Korean literature published in peer-reviewed journals before December 31, 2022 were identified. After review of the 1515 abstracts yielded from the databases and the reference lists of the associated articles, 5 eligible studies were identified, and relevant findings extracted. The five eligible studies will not have found all of the ABR on older adults published in South Korea. However, this shows the level of research and interest in arts-based research in Korea. These five studies had several commonalities. First, they utilized ABR as an extension of qualitative research methods, with arts-based research as another methodology. Second, the distinction between art-based research and qualitative research was not clear in the literature, so art-based research methods were used, but art-based research was not mentioned. Based on these findings, it was suggested that ABR on older adults needs to be revitalized and diversified.

